# PA28γ induces dendritic cell maturation and activates T‐cell immune responses in oral lichen planus

**DOI:** 10.1002/mco2.561

**Published:** 2024-05-08

**Authors:** Yimei Wang, Qiyue Zhang, Xiaoting Deng, Ying Wang, Xin Tian, Shiyu Zhang, Yingqiang Shen, Xikun Zhou, Xin Zeng, Qianming Chen, Lu Jiang, Jing Li

**Affiliations:** ^1^ State Key Laboratory of Oral Diseases, National Clinical Research Center for Oral Diseases, Research Unit of Oral Carcinogenesis and Management, Chinese Academy of Medical Sciences, West China Hospital of Stomatology Sichuan University Chengdu Sichuan PR China; ^2^ Yunnan Maternal and Child Health Hospital Kunming PR China; ^3^ State Key Laboratory of Biotherapy and Cancer Center West China Hospital Sichuan University and Collaborative Innovation Center for Biotherapy Chengdu PR China

**Keywords:** dendritic cell, immune responses, oral lichen planus, PA28γ, T‐cells

## Abstract

Oral lichen planus (OLP) is a common chronic inflammatory disease of the oral mucosa, the mechanism of its inflammatory progression has not yet been fully elucidated. PA28γ plays a significant role in a variety of immune‐related diseases. However, the exact role of PA28γ in the pathogenesis of OLP remains unclear. Here, we demonstrated that PA28γ is overexpressed in epithelial cells and inflammatory cells of OLP tissues but has no significant relationship with OLP subtypes. Functionally, keratinocytes with high PA28γ expression could induce dendritic cell (DC) maturation and promote the T‐cell differentiation into Th1 cells in response to the immune response. In addition, we found that a high level of PA28γ expression is associated with high numbers of infiltrating mature DCs and activated T‐cells in OLP tissues. Mechanistically, keratinocytes with high PA28γ expression could promote the secretion of C–C motif chemokine (CCL)5, blocking CCL5 or/and its receptor CD44 could inhibit the induction of T‐cell differentiation by keratinocytes with high PA28γ expression. In conclusion, we reveal that keratinocytes with high expression of PA28γ in OLP can induce DC maturation and promote T‐cell differentiation through the CCL5‐CD44 pathway, providing previously unidentified mechanistic insights into the mechanism of inflammatory progression in OLP.

## Graphical abstract

Abnormally high expression of PA28γ in oral lichen planus could promote the maturation of dendritic cells and promote the differentiation of T‐cells via the CCL5‐CD44 pathway.

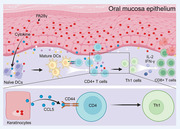

## 1 INTRODUCTION

Oral lichen planus (OLP) is a chronic immune‐mediated inflammatory disease that results in distinctive relapses and remissions and is potentially malignant to the oral mucosa.^1^ The worldwide prevalence of OLP is 1.01%.^2^ The malignant transformation rate is approximately 0.07%–5.8%.^3^, ^4^ Clinically, OLP can be divided into erosive OLP and nonerosive OLP. Erosive OLP is characterized by erythema and ulceration, sometimes with a reticular form, and is generally accompanied by spontaneous pain or a burning sensation; nonerosive OLP is often found by white striae, slightly raised plaques or papules without conscious symptoms or mild irritation pain. In view of the suffering caused by OLP and its own malignant transformation, researchers have focused much of their exploration on its pathogenesis. However, the causes that initiate and/or perpetuate OLP are still not fully understood, and only a small number of risk factors, such as the immune response, genetic background, infectious agents, stress, and drugs, are currently believed to possibly play a role in OLP pathogenesis.^5^ The current literature indicates that immune factors may be key to the pathogenesis of OLP.^6^, ^7^ In particular, T‐cells have been shown to be crucial in the development of illness.^8^ In addition, in the early stage of disease development, more apoptotic keratinocytes can be found,^9^ indicating that keratinocytes may be target cells in the early stage of disease occurrence. Once keratinocytes are activated, they can secrete a variety of inflammatory mediators, including C–C motif chemokine (CCL)5, tumor necrosis factor (TNF)‐α, and interleukin (IL)−1β; recruit inflammatory cells to mediate the development of OLP; amplify inflammatory signals; and promote the chronic progression of OLP.^10^, ^11^ Because the pathogenesis of OLP is unknown, a permanent cure is unavailable. The symptoms of OLP have been treated and managed using a variety of treatment plans, including the elimination of local irritants, topical medications, laser therapy (using carbon dioxide and a low‐dose excimer 308‐nm laser), and systemic therapy; however, the effectiveness of these treatments still needs to be further proven. Therefore, it is imperative to investigate the pathogenesis of OLP.

Proteasome activator complex subunit 3 (PA28γ), often referred to as REGγ, PSME3, Ki antigen, or 11S, is a proteasome activator family member.^12^ Moreover, PA28γ may play a role in intricate cell signaling pathways and immunological responses that contribute to the development of a number of illnesses.^13^ According to our prior study, the expression of PA28γ in OLP tissues is markedly greater than that in normal control tissues,^14^ but the biological role and mechanism of action of PA28γ in OLP are unknown. Dendritic cells (DCs) play a key role in the pathogenesis of OLP by inducing the activation and differentiation of naïve T‐cells through the presentation of antigens to T‐cells.^15^ Previous studies have shown that DCs change from immature to mature in the OLP epithelium and subsequently migrate to the submucosa. This maturation process of DCs is an important immunopathological feature of OLP.^16^ Zhou et al.^17^ previously reported that DCs are impacted by PA28γ, which further regulates the development of Th17 cells, which in turn causes the development of encephalomyelitis, an autoimmune disease. However, whether PA28γ, which is highly expressed in OLP, is involved in the effects of OLP on DCs is unclear. Herein, we found that high PA28γ expression was positively correlated with epithelial and inflammatory cells in OLP patients. Keratinocytes overexpressing PA28γ can promote DC maturation and T‐cell differentiation via the CCL5‐CD44 pathway.

## 2 RESULTS

### 2.1 Abnormal expression of PA28γ in OLP tissues

**FIGURE 1:**
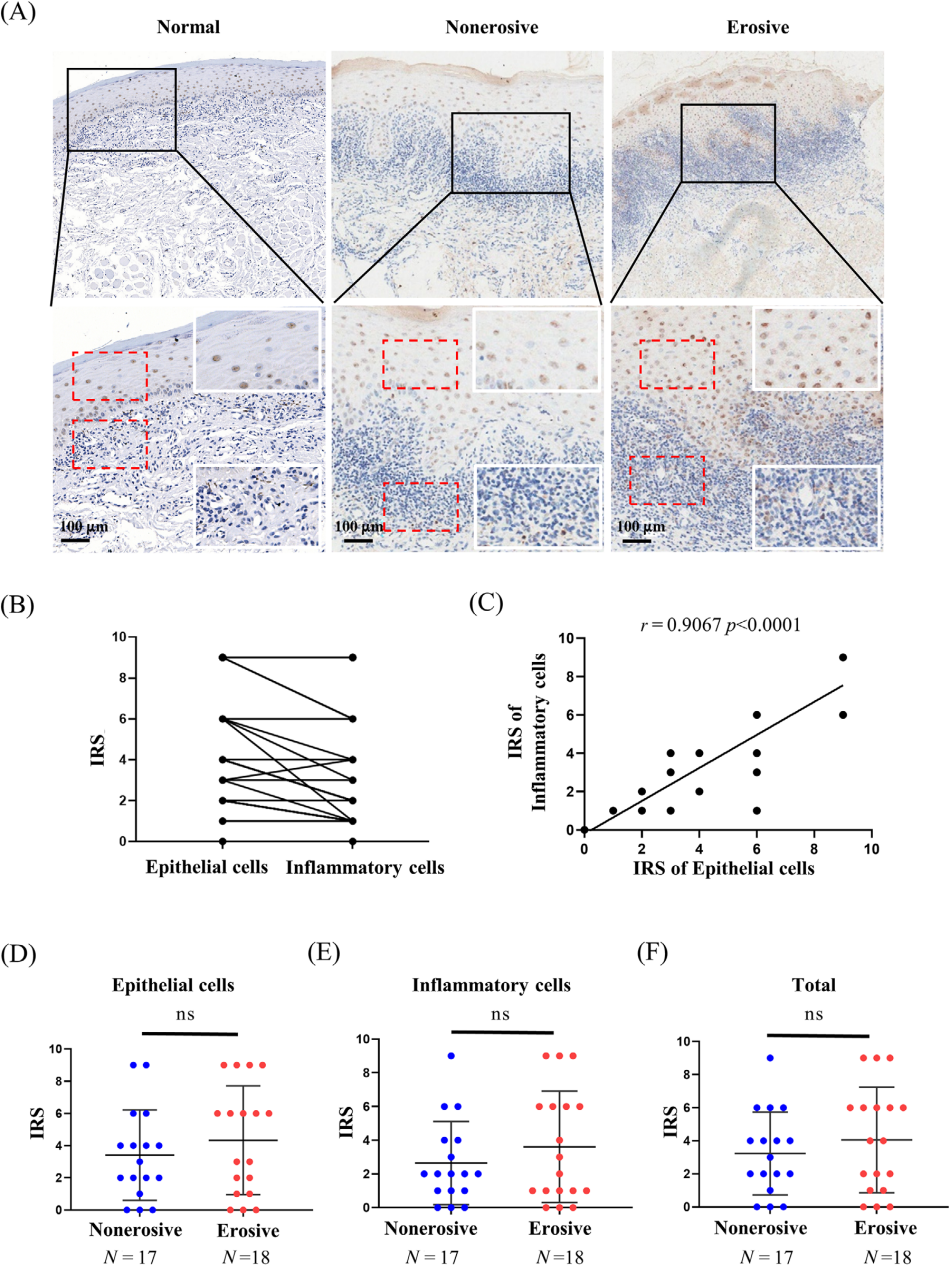
Expression of PA28γ in oral lichen planus (OLP) tissues. (A) Immunohistochemical staining images of PA28γ in normal human oral mucosa and in erosive and nonerosive OLP tissues. The scale bar represents 100 µm. (B, C) Correlation of PA28γ expression in epithelial cells and inflammatory cells (*r* = 0.9067, *p* < 0.0001). (D–F) Statistical analysis of the relationship between PA28γ expression and OLP typing. (D) Relationship between PA28γ expression and OLP typing in epithelial cells. (E) Relationship between PA28γ expression and OLP typing in inflammatory cells. (F) Relationship between PA28γ expression and OLP typing in total cells. IRS, immunoreactivity score; ns = not significant; *p < 0.05; **p < 0.01; ***p < 0.001.

Our initial investigation indicated an upregulation of PA28γ expression in OLP tissues compared to normal controls. However, the functional significance of PA28γ in OLP remains elusive. We conducted immunohistochemistry (IHC) analysis to characterize the expression pattern of PA28γ in erosive and nonerosive OLP tissues. A total of 17 nonerosive OLP and 18 erosive OLP samples were examined. Notably, PA28γ was detected in both epithelial cells and inflammatory cells within the lamina propria (Figure 1A). Linear correlation analysis confirmed a significant positive association between PA28γ expression and both epithelial and inflammatory cells (Figure 1B,C). Interestingly, PA28γ expression levels were higher in epithelial cells compared to inflammatory cells, suggesting that epithelial cells may contribute to the expression of PA28γ in inflammatory cells. Additionally, we investigated the relationship between PA28γ expression and OLP subtypes. However, no significant differences in PA28γ expression were observed among epithelial cells, inflammatory cells, or total OLP tissues between erosive and nonerosive OLP groups (Figure 1D–F). These findings underscore the abnormal upregulation of PA28γ in both epithelial and inflammatory cells within OLP tissues, regardless of OLP subtype.

### 2.2 Induction of dendritic cell maturation and T‐cell activation by keratinocytes overexpressing PA28γ

**FIGURE 2:**
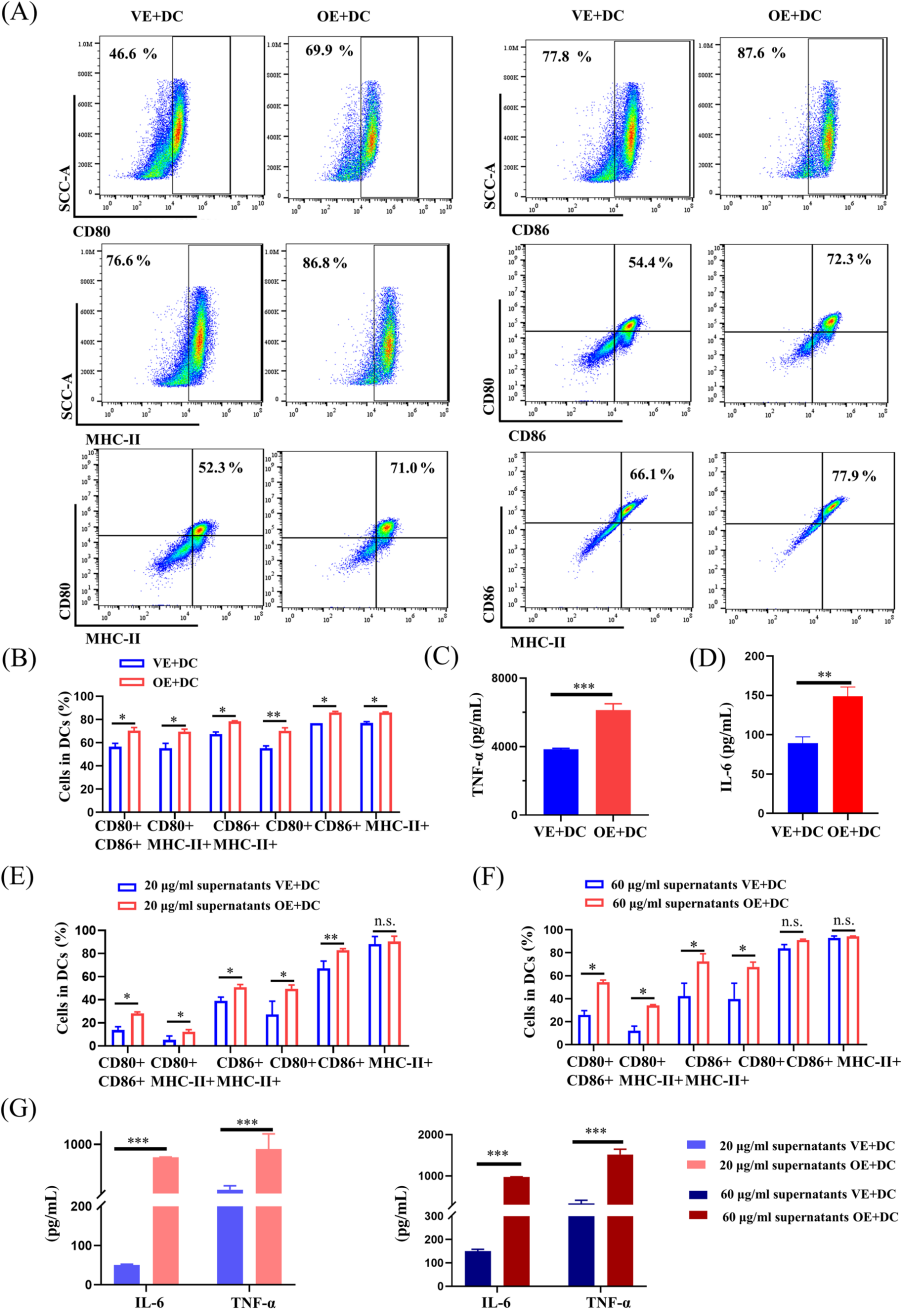
Keratinocytes overexpressing PA28γ induces dendritic cell (DC) maturation. (A) Expression of CD80, CD86, and MHC‐II in DCs after coculture with keratinocytes overexpressing PA28γ. (B) Percentages of DCs that are positive for any one or two of CD80, CD86, and MHC‐II after DCs were cocultured with HaCaT‐OE or HaCaT‐VE cells. (C, D) Tumor necrosis factor (TNF)‐α and interleukin (IL)‐6 levels in the supernatants of DCs cocultured with HaCaT‐OE or HaCaT‐VE cells. (E, F) The percentages of DCs positive for either one or both of CD80, CD86, and MHC‐II after treated with 20 µg/mL or 60 µg/mL HaCaT‐OE or HaCaT‐VE supernatant for 24 h. (G) Enzyme‐linked immunosorbent assay (ELISA) results for IL‐6 and TNF‐α after DCs were treated with 20 µg/mL or 60 µg/mL HaCaT‐OE or HaCaT‐VE supernatant for 24 h. The data are expressed as the mean ± SD and are representative of one experiment with three independent biological replicates; ns = not significant; *p < 0.05; **p < 0.01; ***p < 0.001.

We sought to investigate the potential role of PA28γ in mediating the recruitment of inflammatory cells by keratinocytes in OLP pathogenesis. Human keratinocytes with (HaCaT‐OE) or without (HaCaT‐VE) PA28γ overexpression were cocultured with human monocyte‐derived DCs using Transwell chambers (Figures 2A and S1). Our results revealed that DCs cocultured with supernatants from HaCaT cells overexpressing PA28γ exhibited significantly enhanced expression of MHC‐II, CD86, and CD80 compared to the control group (Figure 2B), indicating the induction of DC maturation by PA28γ‐overexpressing keratinocytes. Additionally, the maturation and activation of DCs led to increased expression of inflammation‐related genes such as TNF‐α and IL‐6,^18^ which play pivotal roles in subsequent immune responses. Consistent with these findings, enzyme‐linked immunosorbent assay (ELISA) analysis demonstrated significantly higher levels of TNF‐α and IL‐6 in DCs cocultured with PA28γ‐overexpressing HaCaT cells compared to controls (Figure 2C,D), confirming the ability of PA28γ‐overexpressing keratinocytes to promote DC maturation and activation. We postulate that epithelial cells overexpressing PA28γ modulate the maturation and activation of DCs by secreting cytokines into the supernatant. To further validate this hypothesis, supernatants from HaCaT cells overexpressing PA28γ and control cells were collected and cocultured with DCs for 24 h. Flow cytometry analysis of DC surface markers confirmed the stimulatory effect of supernatants from PA28γ‐overexpressing HaCaT cells on DCs (Figure 2E,F). Moreover, ELISA analysis revealed increased expression of IL‐6 and TNF‐α in DCs stimulated with supernatants from HaCaT‐OE cells (Figure 2G). These findings underscore the ability of keratinocytes overexpressing PA28γ to induce DC maturation and activation, thereby contributing to T‐cell immune responses in OLP.

**FIGURE 3:**
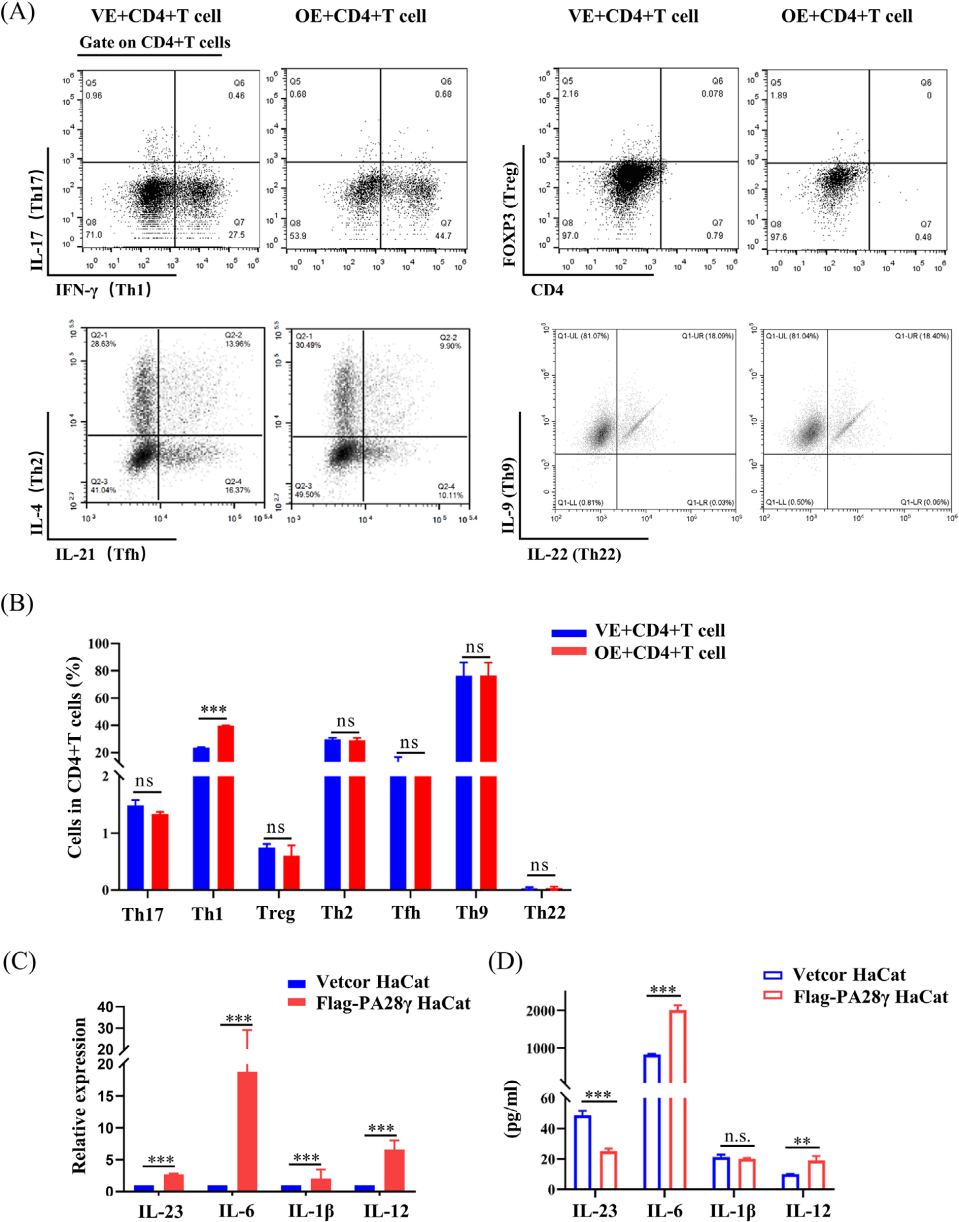
Keratinocytes overexpressing PA28γ activates T‐cell immune responses. (A, B) The expression and statistical percentages of interleukin (IL)‐17, interferon gamma (IFN‐γ), FOXP3, IL‐4, IL‐21, IL‐9, and IL‐22 were detected via flow cytometry after CD4^+^ T‐cells were cocultured with PA28γ‐overexpressing keratinocytes for 24 h. Keratinocytes without PA28γ‐overexpressing were used as a control. (C) IL‐12, IL‐6, IL‐23, and IL‐1β mRNA levels in PA28γ‐overexpressing keratinocytes and control cells. (D) Levels of IL‐12, IL‐6, IL‐23, and IL‐1β in supernatants of PA28γ‐overexpressing keratinocytes and control cells. The data are expressed as the mean ± SD and are representative of one experiment with three independent biological replicates; ns = not significant; *p < 0.05; **p < 0.01; ***p < 0.001.

The interaction between DCs and oral keratinocytes plays a crucial role in T‐cell activation and differentiation. The induction of oral lesions following the local transfer of activated CD4^+^ T‐cell clones underscores the pivotal role of T‐lymphocytes in OLP pathogenesis.^19^ To investigate the impact of PA28γ‐overexpressing keratinocytes on T‐cell responses, we cocultured CD4^+^ T‐cells with HaCaT cells overexpressing PA28γ for 24 h and assessed the expression of various cytokines, including IL‐17, interferon gamma (IFN‐γ), FOXP3, IL‐4, IL‐21, IL‐9, and IL‐22. Notably, the proportion of Th1 cells (IFN‐γ) was significantly higher in the overexpression group compared to the control group (Figure 3A,B), suggesting a potential role of PA28γ in promoting Th1 cell differentiation. Furthermore, we hypothesized that PA28γ‐overexpressing keratinocytes might induce further differentiation of naïve CD4^+^ T‐cells through the secretion of cytokines. Our findings revealed that HaCaT cells overexpressing PA28γ exhibited increased mRNA levels of IL‐1β, IL‐23, IL‐6, and IL‐12 compared to control keratinocytes (Figure 3C). Consistently, the protein levels of IL‐6 and IL‐12 in the supernatant of PA28γ‐overexpressing HaCaT cells were also elevated (Figure 3D). These results suggest that PA28γ‐overexpressing keratinocytes have the capacity to secrete cytokines that facilitate the differentiation of CD4^+^ T‐cells into Th1 and Th17 cells, thereby promoting T‐cell immune responses. Notably, IL‐12 has been reported to promote the differentiation of Th1 cells,^20^ while IL‐6, IL‐23, and IL‐1 facilitate the differentiation and function of Th17 cells.^21^ Thus, the upregulation of these cytokines in PA28γ‐overexpressing keratinocytes may contribute to the activation and polarization of T‐cell responses in OLP.

### 2.3 Association between high PA28γ expression and infiltration of mature DCs in OLP tissues

**FIGURE 4:**
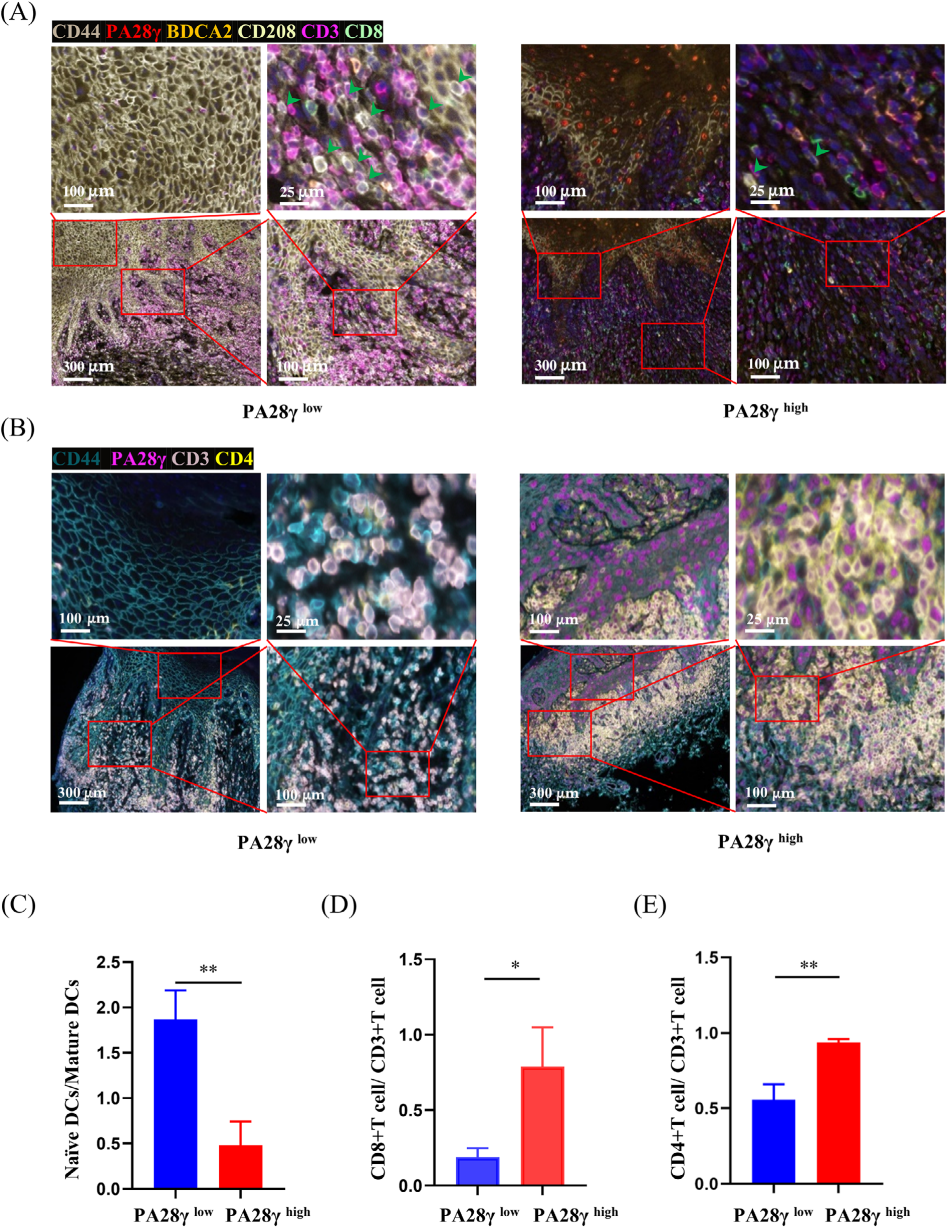
PA28γ promotes dendritic cell (DC) maturation and T‐cell responses in oral lichen planus (OLP) tissues. (A–E) Multiplex consecutive immunohistochemical staining of OLP tissue with high and low expression of PA28γ and corresponding statistical analysis. (A, B) Multiplex consecutive immunohistochemical staining of OLP tissue. The scale bars represent 300 μm, 100 μm, and 25 μm. The green arrows represent naïve DCs. (C–E) Statistical analysis. The data are expressed as the mean ± SD and are representative of one experiment with three independent biological replicates; ns = not significant; *p < 0.05; **p < 0.01; ***p < 0.001.

Multiplex immunofluorescence (mIHC) analysis was employed to delineate the cellular composition of OLP samples. These samples were stratified into two groups based on PA28γ expression levels: keratinocytes with high PA28γ expression and keratinocytes with low PA28γ expression. Results demonstrated that OLP tissues exhibiting high PA28γ expression displayed an increased presence of mature DCs and a decreased abundance of naïve DCs, whereas OLP tissues with low PA28γ expression showed the opposite trend (Figure 4A). Subsequent statistical analysis revealed a higher density of mature DCs and CD8^+^ T‐cells in OLP tissues with elevated PA28γ expression (Figure 4C,D). Similarly, analysis of another panel demonstrated a greater number of CD4^+^ T‐cells in OLP tissues with high PA28γ expression (Figure 4B,E). These findings underscore the role of keratinocytes with high PA28γ expression in promoting the maturation of DCs, thus enhancing their antigen‐presenting capacity and facilitating the activation of T‐cell responses.

### 2.4 Contribution of keratinocytes overexpressing PA28γ to CD4+ T‐cell differentiation via the CCL5‐CD44 pathway in OLP

**FIGURE 5:**
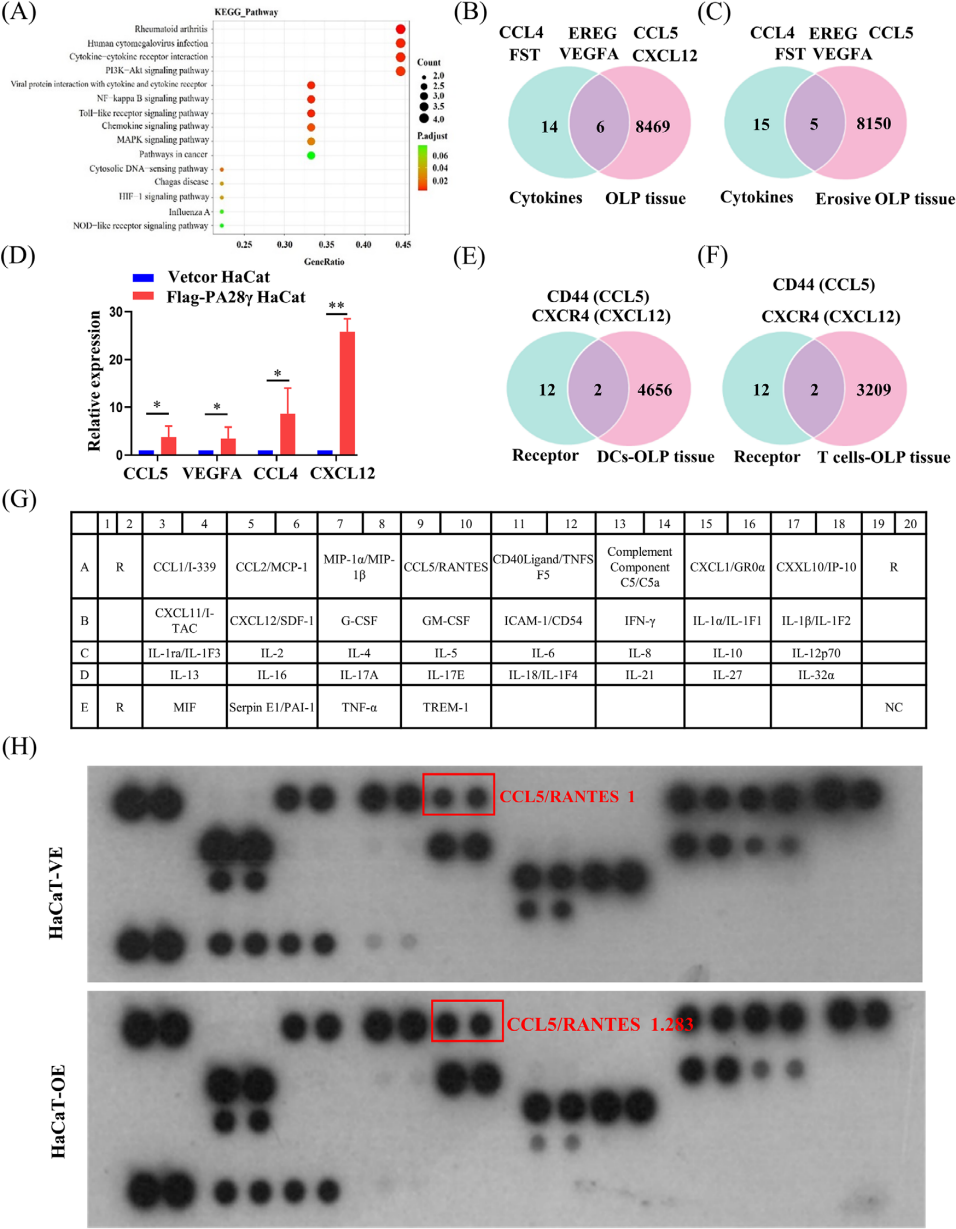
Keratinocytes overexpressing PA28γ may promote CD4^+^ T‐cell differentiation through the CCL5‐CD44 pathway. (A) Kyoto Encyclopedia of Genes and Genomes (KEGG) pathway analysis of cytokine microarray results of P HaCaT cells overexpressing PA28γ cocultured with the vector. (B, C) Analysis of cytokine chip and single‐cell sequencing data for oral lichen planus (OLP) normal tissue epithelial cells and erosive–nonerosive OLP epithelial cells. (D) The mRNA expression levels of CCL5, vascular endothelial growth factor A (VEGFA), CCL4, and CXCL12 in HaCaT cells overexpressing PA28γ and control cells. (E, F) The expression of the CXCL12, CCL5, VEGFA, and CCL4 receptors was analyzed with respect to the gene expression data of dendritic cells (DCs; E) and T‐cells (F) in OLP from the previous single‐cell sequencing data of the research group, and a Venn diagram was generated for the two shared receptors. (G) Schematic representation of cytokine and chemokine antibodies present on a cytokine antibody array. (H) Human cytokine array analysis of supernatants from HaCaT‐VE and HaCaT‐OE. The numerical value shows the ratio of the C–C motif chemokine (CCL)5 gray value of the two groups to the CCL5 gray value of HaCaT‐VE. The data are expressed as the mean ± SD and are representative of one experiment with three independent biological replicates; ns = no significant; *p < 0.05; **p < 0.01; ***p < 0.001.

We have established that PA28γ can stimulate the maturation of DCs and promote the differentiation of CD4^+^ T‐cells. However, the precise mechanism underlying this process remains elusive. Therefore, we hypothesized that PA28γ might influence the secretion of cytokines by keratinocytes, thereby modulating the function of DCs and CD4^+^ T‐cells in conjunction with cytokine receptors. To investigate this hypothesis, we collected culture supernatants from keratinocytes with or without PA28γ overexpression and analyzed the expression levels of over 500 cytokines associated with T‐cell differentiation and chemotaxis. Our analysis revealed differential expression of 20 cytokines, with 10 being upregulated and 10 downregulated (Table S1). Additionally, Kyoto Encyclopedia of Genes and Genomes pathway analysis indicated that differentially expressed genes were primarily linked to inflammatory pathways, including nuclear factor kappa B, Toll‐like receptor, mitogen‐activated protein kinase, and PI3K/Akt signaling pathways (Figure 5A). Furthermore, we integrated single‐cell RNA sequencing (scRNA‐seq) data of epithelial cells from OLP tissues with cytokine data using Venn diagram software, screening‐specific cytokines associated with OLP (Figure 5B,C). Subsequent validation via quantitative polymerase chain reaction (qPCR) confirmed significantly higher mRNA expression levels of CCL5, vascular endothelial growth factor A (VEGFA), CCL4, and CXCL12 in the PA28γ overexpression group compared to the control group (Figure 5D). Notably, the receptors for these cytokines, namely, CXCR4, CXCR7, ACKR3, CD44, CCR5, GPR75, CCR1, CCR3, VEGFR1, VEGFR2, CCR1, and CCR5, were analyzed alongside gene expression data of DCs and T‐cells in OLP tissue. Interestingly, CD44 and CXCR4 emerged as overlapping receptors (Figure 5E,F). Moreover, analysis of The Cancer Genome Atlas (TCGA) database revealed positive correlations between PA28γ and CD44 mRNA expression (Figure S2), suggesting a potential role for PA28γ in facilitating CD4^+^ T‐cell differentiation into Th1 cells via the CCL5‐CD44 pathway. These findings provide insights into the mechanism by which keratinocytes overexpressing PA28γ contribute to CD4^+^ T‐cell differentiation in OLP, shedding light on potential therapeutic targets for modulating immune responses in this condition.

**FIGURE 6:**
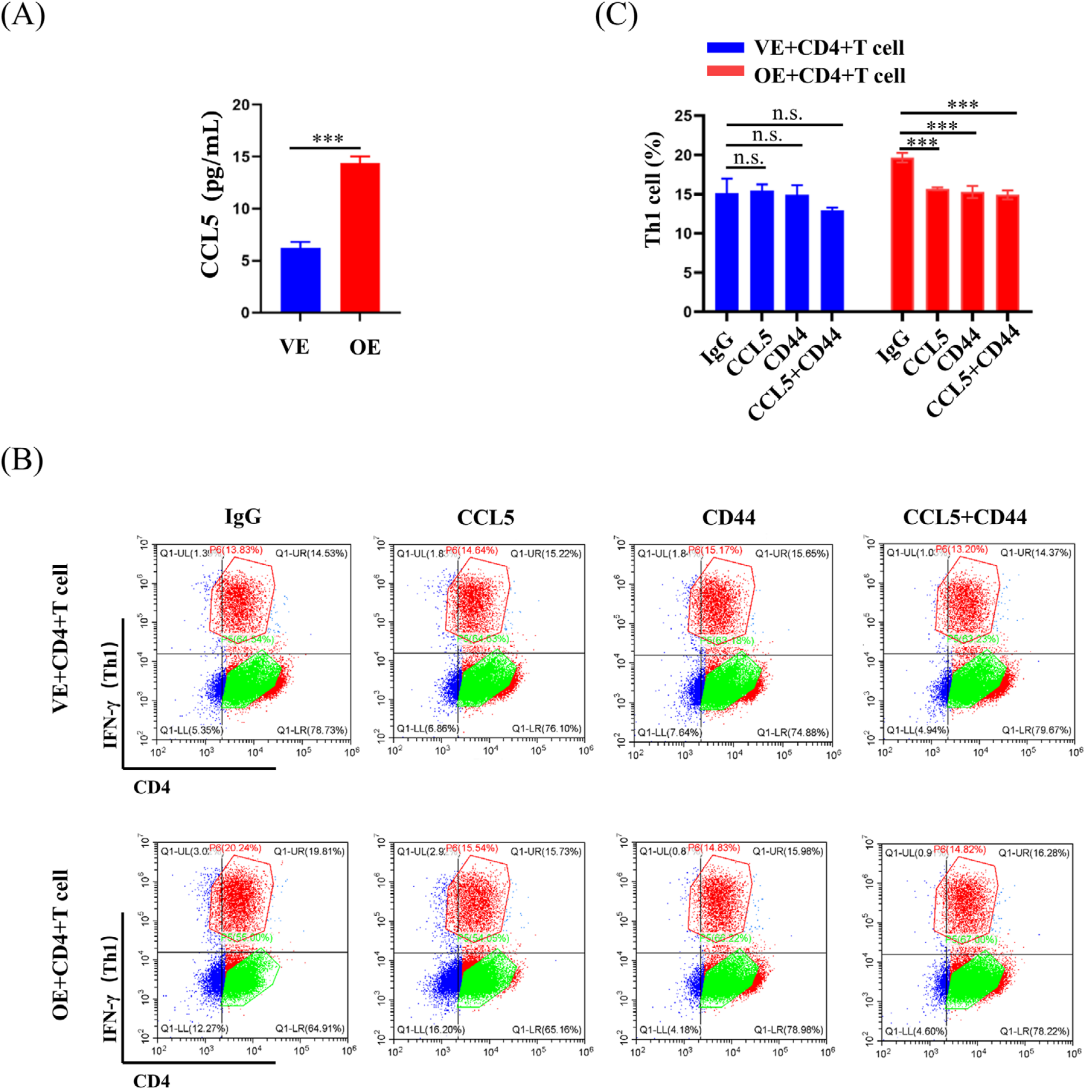
Blocking the CCL5‐CD44 pathway can inhibit CD4^+^ T‐cell differentiation caused by keratinized epithelial cells with high PA28γ expression. (A) Enzyme‐linked immunosorbent assay (ELISA) analysis of CCL5 in the culture supernatant of HaCaT‐OE or HaCaT‐VE cells. (B) Expression of interferon gamma (IFN‐γ) in CD4^+^ T‐cells after stimulation with concentrated supernatants of PA28γ‐overexpressing HaCaT cells and control cells and incubation for 24 h with the addition of CCL5, CD44 neutralizing antibody, or IgG isotype control to the culture medium. (C) IFN‐γ (Th1) positive cell percentage statistics. The data are expressed as the mean ± SD and are representative of one experiment with three independent biological replicates; ns = not significant; *p < 0.05; **p < 0.01; ***p < 0.001.

To further investigate the impact of PA28γ overexpression on cytokine secretion, we conducted a thorough examination of cytokine expression in the supernatants of PA28γ‐overexpressing keratinocytes and control cells using a human cytokine array containing CCL5. The results revealed a significant increase in the level of CCL5 in the supernatant of PA28γ‐overexpressing cells compared to the control group (Figure 5G,H). This finding was further validated by ELISA, which also demonstrated elevated secretion of CCL5 by PA28γ‐overexpressing keratinocytes (Figure 6A). Subsequently, we aimed to elucidate the functional implications of elevated CCL5 secretion by PA28γ‐overexpressing keratinocytes in CD4^+^ T‐cell differentiation. To achieve this, we used supernatants of HaCaT cells overexpressing PA28γ to stimulate CD4^+^ T‐cells and utilized antibodies to neutralize CCL5 and CD44. Following a 24‐h coculture period, the level of IFN‐γ (Th1) was measured by flow cytometry. Remarkably, the results indicated a significant reduction in the level of IFN‐γ after the introduction of monoclonal antibodies targeting CCL5 or/and CD44 (Figure 6B,C). These findings strongly suggest that keratinocytes overexpressing PA28γ can enhance the differentiation of CD4^+^ T‐cells into Th1 cells through the CCL5‐CD44 pathway, highlighting the critical role of PA28γ in modulating immune responses in the context of OLP.

## 3 DISCUSSION

Despite being recognized as a common oral potentially malignant disorder and a T‐cell‐mediated autoimmune disease, the pathogenic mechanisms and precipitating factors of OLP remain poorly understood. Currently, the main focus in clinical management revolves around symptom alleviation and treatment options such as corticosteroids, calcineurin inhibitors, and photodynamic therapy. Among these, topical steroids have emerged as a cost‐effective and efficient treatment modality,^22^, ^23^ with combination therapies yielding even better outcomes. For instance, the combination of acitretin and triamcinolone acetonide has demonstrated superior efficacy compared to triamcinolone acetonide monotherapy.^24^ However, due to the unclear pathogenic mechanisms, OLP often recurs, leading to pain and anxiety among patients, thus significantly impacting their quality of life. In this study, we identified a potential link between keratinocytes expressing high levels of PA28γ and the maturation of DCs, along with the elicitation of T‐cell immune responses via the CCL5‐CD44 pathway. These findings not only contribute to our understanding of OLP pathogenesis but also offer new avenues for its treatment.

Functional investigations have highlighted the involvement of PA28γ in various cellular processes, including the immune response, cell signaling, cell cycle regulation, and apoptosis.^12^ Our previous observations of increased PA28γ expression in OLP tissues led us to speculate that PA28γ might influence OLP development by modulating the immune system. Given the pivotal role of T‐cells in OLP development, particularly CD8 tissue‐resident memory T‐cells that secrete cytokines promoting OLP erosion,^25^ as well as the induction of oral lesions by activated CD4^+^ T‐cell clones,^19^ and DCs are important antigen‐presenting cells, play a role in the development of the majority of autoimmune illnesses during the immune response, we hypothesized that PA28γ in OLP may indeed influence DCs and subsequent T‐cell immune responses, thereby impacting disease progression.

We investigated the expression pattern of PA28γ in OLP and observed its presence in both epithelial and lamina propria inflammatory cells, with a positive correlation between its expression levels in these cell types. Previous studies have reported that keratinocytes can release inflammatory mediators to recruit inflammatory cells and promote OLP development.^10^ Given this, we hypothesized that keratinocytes expressing high levels of PA28γ might influence this process. To test this hypothesis, we used lentiviral constructs to overexpress PA28γ in keratinocytes and cocultured them with DCs, followed by flow cytometry analysis. Our results indicated that high levels of PA28γ in keratinocytes increased the maturation of DCs by upregulating the expression of CD80, CD86, and MHC‐II molecules. Mature DCs are crucial for T‐cell differentiation and proliferation.^26^ The supernatant from keratinocytes with high PA28γ expression also promoted the expression of these molecules in DCs, leading to enhanced CD4^+^ T‐cell development and the production of additional pro‐Th1 or Th2 cytokines in response to the costimulatory molecules CD80 and CD86. This facilitates the rapid and efficient identification of antigenic peptides presented by MHC‐II molecules by DCs.

Furthermore, mature DCs release various cytokines, including TNF‐α and IL‐6,^27^ which not only promote T‐cell proliferation and differentiation but also enhance T‐cell adherence by upregulating the expression of adhesion molecules, facilitating T‐lymphocyte infiltration.^28^ TNF‐α stimulation induces the production of chemokines and adhesion molecules, promoting lymphocyte recruitment^1^, ^29^, ^30^, ^31^, ^32^ and leading to breaks in the epithelial basement membrane.^33^, ^34^, ^35^ Additionally, TNF‐α can induce apoptosis in keratinocytes by binding to TNF receptors on their surface.^36^, ^37^ We found that IL‐6 and TNF‐α levels were significantly increased in DCs cocultured with PA28γ‐overexpressing keratinocytes or treated with supernatants from these keratinocytes. Upregulation of TNF‐α and IL‐6 secretion by DCs could subsequently induce lymphocyte aggregation and keratinocyte apoptosis, both contributing to the advancement of OLP. As primary antigen‐presenting cells, DCs play a pivotal role in stimulating the T‐cell immune response, while CD4^+^ T‐cells are central to the pathogenesis and development of OLP. Coculture of CD4^+^ T‐cells with PA28γ‐overexpressing keratinocytes demonstrated an increase in CD4^+^ T‐cell development toward the Th1 phenotype. Further investigation revealed higher mRNA levels of cytokines such as IL‐12, IL‐6, IL‐23, and IL‐1 in PA28γ‐overexpressing keratinocytes compared to control cells, along with elevated protein levels of IL‐12 and IL‐6 in their supernatant. These cytokines are known to promote the development and activity of Th1 and Th17 cells,^38^, ^39^ indicating that PA28γ‐overexpressing keratinocytes can release cytokines associated with the differentiation of CD4^+^ T‐cells into Th1 and Th17 cells, thereby stimulating T‐cell immune responses.

IL‐12 and IFN‐γ are well‐known regulators of CD4^+^ T‐cell differentiation, with IL‐12 activating STAT4 and subsequently influencing CD4^+^ T‐cell fate.^40^ However, to delve deeper into the upstream mechanisms regulating CD4^+^ T‐cell differentiation, we explored the role of PA28γ in this process. Comparative analysis of cytokine microarray results from keratinocytes overexpressing PA28γ and negative controls revealed differential expression of 20 cytokines. Subsequent examination of cytokine microarray and single‐cell sequencing data from epithelial cells in OLP tissues identified six key cytokines, including CCL4, EREG, CCL5, FST, VEGFA, and CXCL12. Further analysis of cytokine microarray and scRNA‐seq data specifically from erosive OLP tissues highlighted five cytokines, aligning with previous findings, with CCL5, VEGFA, CCL4, and CXCL12 emerging as prominent candidates. This led us to investigate cytokine receptors in conjunction with scRNA‐seq data from DCs and T‐cells in OLP tissues, revealing CD44 and CXCR4 as two overlapping receptors. Subsequent validation through human cytokine arrays confirmed that PA28γ overexpression promotes increased expression of CCL5 and IL‐6. Examination of the TCGA database unveiled positive correlations between PA28γ expression and both CD44 and CXCR4 expression in the head and neck squamous cell carcinoma (HNSCC) cohort. These findings suggest that PA28γ may facilitate CD4^+^ T‐cell differentiation into Th1 cells through the CCL5‐CD44 pathway, a hypothesis further supported by antibody neutralization experiments. Additionally, mIHC experiments demonstrated that OLP tissues with high PA28γ expression exhibited greater numbers of mature DCs and CD8^+^ T‐cells compared to those with low PA28γ expression, consistent with previous findings. We speculate that CD8^+^ T‐cells recognize antigen‐associated major histocompatibility complex class I (MHCI) molecules on diseased keratinocytes and are activated by cytokines secreted by Th1 cells, such as IL‐2 and IFN‐γ, leading to keratinocyte apoptosis and OLP development. However, further validation of this hypothesis is warranted.

CD44, a cell surface protein extensively expressed on lymphocytes and nonlymphoid cells, plays crucial roles in tumor angiogenesis, lymphocyte homing, activation, and proliferation, as well as cell adhesion and migration.^41^, ^42^, ^43^, ^44^ Notably, CD44 recruitment during DC–T‐cell contacts impacts T‐cell activation, cytokine production, and immunological synapse formation.^45^ Studies suggest that CD44 deletion promotes Th2 differentiation while suppressing Th1 differentiation,^46^ consistent with our findings.

## 4 CONCLUSION

In conclusion, our study highlights the role of keratinocytes with elevated PA28γ expression in promoting DC maturation through cytokine secretion, consequently eliciting T‐cell immunological responses. Furthermore, we observed that PA28γ facilitated T‐cell differentiation via the CCL5‐CD44 pathway. These findings contribute to a better understanding of the pathogenesis of OLP and suggest potential therapeutic targets for intervention in this condition.

## 5 MATERIALS AND METHODS

### 5.1 Immunohistochemistry

IHC for PA28γ (Thermo Fisher, #PA5‐21789, 1:400) staining of formalin‐fixed paraffin‐embedded (FFPE) samples following antigen extraction with citrate buffer (0.01 M, pH 6.0), followed by staining with diaminobenzidine (DAKO, GK600510, 1:50) for 10 min. This was followed by counterstaining with hematoxylin (Biosharp, 171830) for 30 s, after which the nuclei were visualized. For PA28γ, staining intensity was assessed by two experienced pathologists who lacked clinical and pathological information (0, no staining; 1, weak staining; 3, strong staining; and tan), and the staining range was <10% (grade 1): grade 2: 10%–30%; grade 3: 31%–70%; and grade 4: >70%. The proportion of positive cells was recorded. Immunoreactivity score = staining intensity × staining range.

### 5.2 Cell cultures

The stable overexpression of PA28γ in HaCaT cells (HaCaT‐OE) and HaCaT‐vector cells (HaCaT‐VE) was confirmed by the group's previous work (identification of a BRAF/PA28γ/MEK1 signaling axis in oral submucous fibrosis and its function in epithelial–mesenchymal transition). HaCaT‐OE and HaCaT‐VE cells were cultured in Dulbecco's modified Eagle's medium supplemented with 10% fetal bovine serum (Gibco) and 1% penicillin/streptomycin. All cells were raised at 5% CO_2_ and 37°C.

### 5.3 DC and T‐cell generation

A 2‐h adhesion step was used to separate monocytes from peripheral blood mononuclear cells (PBMCs) at 37°C. Adherent monocytes were treated with a combination of IL‐4 (PeproTech, AF‐200‐04‐20, 20 ng/mL) and granulocyte‐macrophage colony stimulating factor (GM‐CSF) (PeproTech, AF‐300‐03‐20, 20 ng/mL) after nonadherent cells were removed. The cultures were incubated in 1640 medium (HyClone) supplemented with 10% fetal bovine serum. Every 48 h, half‐volume adjustments were made, and fresh 20 ng/mL IL‐4 and 20 ng/mL GM‐CSF supplements were added; the cells were cultured for 7 days to obtain DCs. Afterward, the cell purity was determined by flow cytometry; additionally, the isotype control served as the adverse control.

PBMCs were collected using density gradient centrifugation, as previously described, and CD4^+^ T‐cells were subsequently purified using antihuman CD4 MicroBeads on a magnetic activated cell sorting (MACS) magnetic rack (Miltenyi Biotec). Purified CD4^+^ T‐cells were resuspended in 1640 medium (HyClone) containing 10% fetal bovine serum (Gibco) at a concentration of 1 × 10^6^/mL. Pure mouse antihuman CD3 antibody (BD Pharmingen, 553057, 10 μg/mL) and purified mouse antihuman CD28 antibody (BD Pharmingen, 553294, 5 μg/mL) were added to the mixture to activate CD4^+^ T‐cells.

### 5.4 RNA isolation and qRT‐PCR

Using RNA Pure kits (Zymo Research, TR‐205‐50), total RNA was extracted in accordance with the manufacturer's instructions. The concentration and purity of the extracted RNA were assessed using a NanoDrop Microvolume UV/Vis Spectrophotometer (Thermo Fisher Scientific). To produce the necessary complementary DNA, the extracted RNA was then reverse transcribed using the PrimeScriptTM RT Reagent Kit (TaKaRa, RR037A) in accordance with the kit's instructions. SYBR™ Select Master Mix (Applied Biosystems, 4472908) was used to create a 20 μL total volume of the real‐time (RT)‒PCR system in accordance with the manufacturer's recommendations. RT‒PCR was carried out using a QuantStudio 3 Real‐Time Fluorescence PCR System (Applied Biosystems) on the samples to be analyzed. The relative expression of mRNA in the cells was determined using the 2−ΔΔCt method. The data of three biological duplicate experiments are shown in these histograms. All the data are presented as the mean ± SD of three experiments. In Table S2, a list of primer sequences is provided. For each reaction, three technical duplicates were performed to guarantee reliability and validity. The CT value of the target gene was calculated and compared to that of the reference gene glyceraldehyde 3‐phosphate dehydrogenase (GAPDH).

### 5.5 Western blot analysis

Using radio immunoprecipitation assay lysis buffer, cell protein was extracted. The primary antibodies used for Western blot analysis were Flag‐Tag (Sigma‒Aldrich, F1804, 1:1000), PA28γ (Invitrogen, PA5‐21789, 1:2000), GAPDH (Cell Signaling Technology, 5174S, 1:1000).

### 5.6 Flow cytometry

DCs were collected and counted, and 1 × 10^6^ cells were suspended in phosphate buffered saline (200 μL) and stained with MHC‐II‐BV510, anti‐CD209‐PE, anti‐CD80‐APC, and anti‐CD86‐BV605 antibodies from BioLegend. CD4^+^ T‐cells were collected and counted, and 1 × 10^6^/mL cells were incubated in 1640 medium for 4 h and then suspended in a solution containing 50 ng/mL phorbol 12‐myristate 13‐acetate (PMA), 1 μg/mL ionomycin, and 0.1% Golgi blocker. The cells were first stained with antihuman CD4‐FITC and antihuman CD8‐APC antibodies from BioLegend for 30 min and subsequently successively fixed using Cytofix/Cytoperm in accordance with the manufacturer's instructions. After 30 min of staining, the cells were fixed and permeabilized using a Cytofix/Cytoperm solution kit (BD Biosciences) in accordance with the manufacturer's instructions. Next, antihuman IFNγ‐PE, antihuman IL‐4 APC, antihuman IL‐9 PE/Cyanine7, antihuman IL‐21 PE, antihuman IL‐22 FITC, and antihuman IL‐17 APC/CY7 antibodies (BioLegend) were used to stain the cells. FlowJo software and an Attune®NxT flow cytometer were used to assess the stained cells. The statistical analysis findings disregarded the impact of the isotype control.

### 5.7 ELISA

Using ELISA kits, the cytokine concentrations in the culture media were evaluated in triplicate. IL‐6 (Neobioscience, RK00004), IL‐12 (Jianglai Biological, JL18332‐48T48T), CCL5 (Ruixin Biology, RX104824H), IL‐23 (Ruixin Biology, RX106146H), Il‐1β (Ruixin Biology, RX106152H), and TNF‐α (Fine Biotech, EH4080) ELISA kits were used.

### 5.8 Cytokine chip detection

The culture supernatants of HaCaT‐OE and HaCaT‐VE cells were collected, and Wayen Biotechnologies (Shanghai), Inc. tested the expression levels of more than 500 cytokines related to T‐cell differentiation and chemotaxis.

### 5.9 Analysis of human cytokine array

HaCaT‐OE and HaCaT‐VE cells were cultured normally according to the above conditions, and the supernatants were subsequently harvested after 48 h. Human cytokine array was used to identify the supernatants (R&D Systems, ARY005).

### 5.10 Multiplex immunohistochemistry

mIHC staining of FFPE OLP samples was performed using the Panovue 7‐color kit (Cat#10004100100) according to the manufacturer's instructions. Primary antibodies against CD44 (Abcam, ab189525, 1:2000), CD3 (Abcam, ab16669, 1:500), CD4 (Abcam, ab133616, 1:500), CD8 (Abcam, ab245118, 1:1000), CD208 (Abcam, ab271053, 1:500), BDCA2 (Abcam, ab239078, 1:1000), and PA28γ (Invitrogen, PA5‐21789, 1:500) were used. 4ʹ,6‐diamidino‐2‐phenylindole (Sigma‒Aldrich) was used as the final stain. Using Inform software, naïve DCs, mature DCs, and CD4^+^ and CD3^+^ T‐cells were manually chosen for quantitative analysis.

### 5.11 Antibody neutralization

We used concentrated supernatants of HaCaT cells overexpressing PA28γ to stimulate CD4^+^ T‐cells, and CCL5, CD44 neutralizing antibodies or immunoglobulin G (IgG) isotype controls were added to the culture medium and incubated for 24 h before flow cytometry detection was performed. For CCL5 (R&D, AF‐278‐NA) and CD44 (Abcam, ab189524), the concentration was 10 times the corresponding neutralizing dose 50 (ND50).

### 5.12 Bioinformatics analysis

The scRNA‐seq data of OLP were obtained from the Gene Expression Omnibus (GEO) database (GSE211630). The correlations of CD44, CXCR4, and PA28γ mRNA expression in the HNSCC cohort were analyzed through the TCGA database (http://gdac.broadinstitute.org).

### 5.13 Statistical analysis

GraphPad Prism software was used to statistically evaluate all the data, and the mean ± SD of three experiments was used to express the data for each group. Independent sample *t*‐tests were used for statistical analysis. Differences were considered to be statistically significant at *p* < 0.05. *p* values <0.05 were considered to indicate statistical significance. Significant differences are expressed as *p < 0.05, **p < 0.01, and ***p < 0.001.

## AUTHOR CONTRIBUTIONS

J.L. and L.J. designed the experiment. Y.W., Q.Z., and X.D. performed the experiments. Y.W., Q.Z., X.D., S.Z., X.T., Y.W., Y.S., X.Z., X.Z., and Q.C. analyzed the data and drafted the manuscript. All the authors read and approved the final version of the manuscript.

## CONFLICT OF INTEREST STATEMENT

The authors declare no conflicts of interest.

## ETHICS STATEMENT

Tissue samples were collected from patients diagnosed with OLP (Table S3) and blood samples were obtained from healthy individuals. Informed consent was obtained from all participants to collect OLP tissue and blood samples for the study. The Ethics Committee of West China Stomatological Hospital of Sichuan University (WCHSIRB‐D‐2022‐021) approved this study.

## Supporting information

Supporting Information

Supporting Information

## Data Availability

The scRNA‐seq data of OLP were obtained from the GEO database (GSE211630). All relevant data supporting the conclusions of this article are included within the article.
