# Supplementary material for: PA28γ induces dendritic cell maturation and activates T‐cell immune responses in oral lichen planus
Source: MedComm (2020). 2024 May 8;5(5):e561. doi: 10.1002/mco2.561 (PMC11077662; doi:10.1002/mco2.561)

**PA28γ Induces Dendritic Cell Maturation and Activates T-cell Immune Responses in Oral Lichen Planus**

**Yimei Wang^1,#^, Qiyue Zhang^1,#^, Xiaoting Deng^2,#^, Ying Wang^1^, Xin Tian^1^, Shiyu Zhang^1^, Yingqiang Shen^1^, Xikun Zhou^3^, Xin Zeng^1^, Qianming Chen^1^, Lu Jiang^1,*^, Jing Li^1,*^**

^1^State Key Laboratory of Oral Diseases, National Clinical Research Center for Oral Diseases, Research Unit of Oral Carcinogenesis and Management, Chinese Academy of Medical Sciences, West China Hospital of Stomatology, Sichuan University, Chengdu, Sichuan, 610041, P. R. China.

^2^Yunnan Maternal and Child Health Hospital, Kunming 650000, P. R. China.

^3^State Key Laboratory of Biotherapy and Cancer Center, West China Hospital, Sichuan University and Collaborative Innovation Center for Biotherapy, Chengdu 610041, P. R. China.

# These authors contributed equally to this work and share first authorship.

* To whom correspondence should be addressed:

Corresponding Author

Jing Li

State Key Laboratory of Oral Diseases, National Clinical Research Center for Oral Diseases, West China Hospital of Stomatology, Sichuan University

Email: [lijing1984@scu.edu.cn](mailto:lijing1984@scu.edu.cn)

Lu Jiang

State Key Laboratory of Oral Diseases, National Clinical Research Center for Oral Diseases, West China Hospital of Stomatology, Sichuan University

Email: [jianglu@scu.edu.cn](mailto:jianglu@scu.edu.cn)


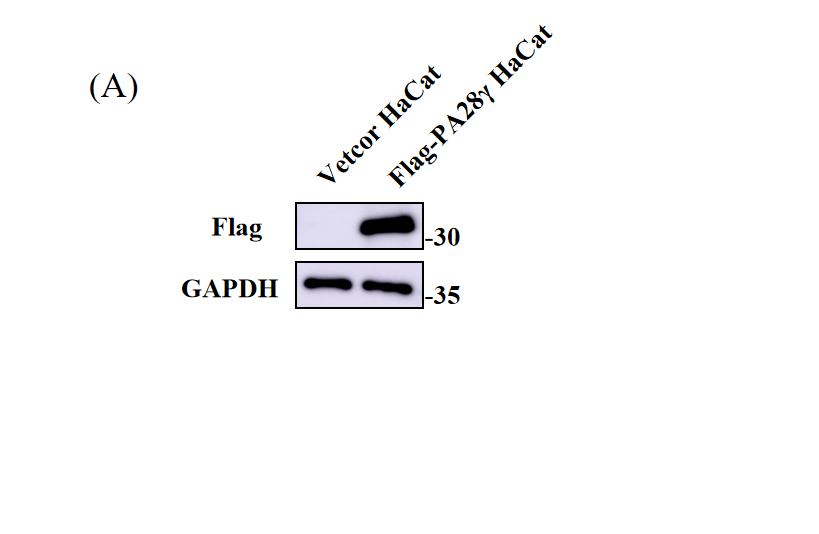


**Figure S1 Keratinocytes have overexpressed PA28γ**

Western blot analysis of PA28γ-overexpressing HaCaT cells and control cells.


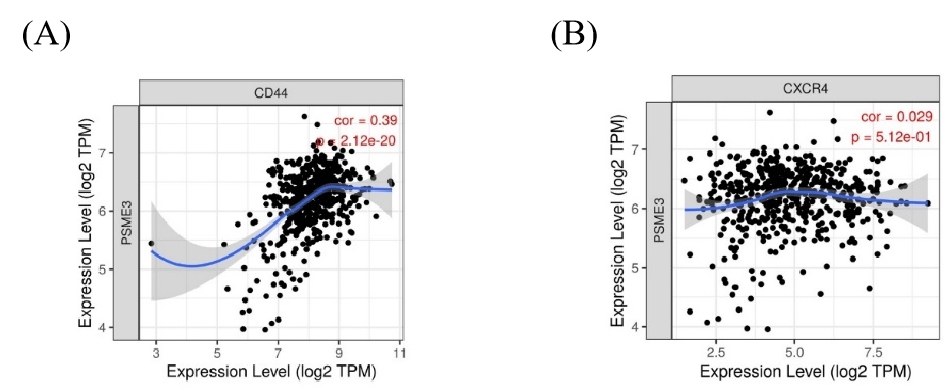


**Figure S2 Correlation analysis of CD44, CXCR4 and PA28γ**

(A, B) The correlations of CD44, CXCR4 and PA28γ mRNA expression in the head and neck squamous cell carcinoma (HNSCC) cohort were analyzed through the TCGA database.

**Table S2. List of primer**


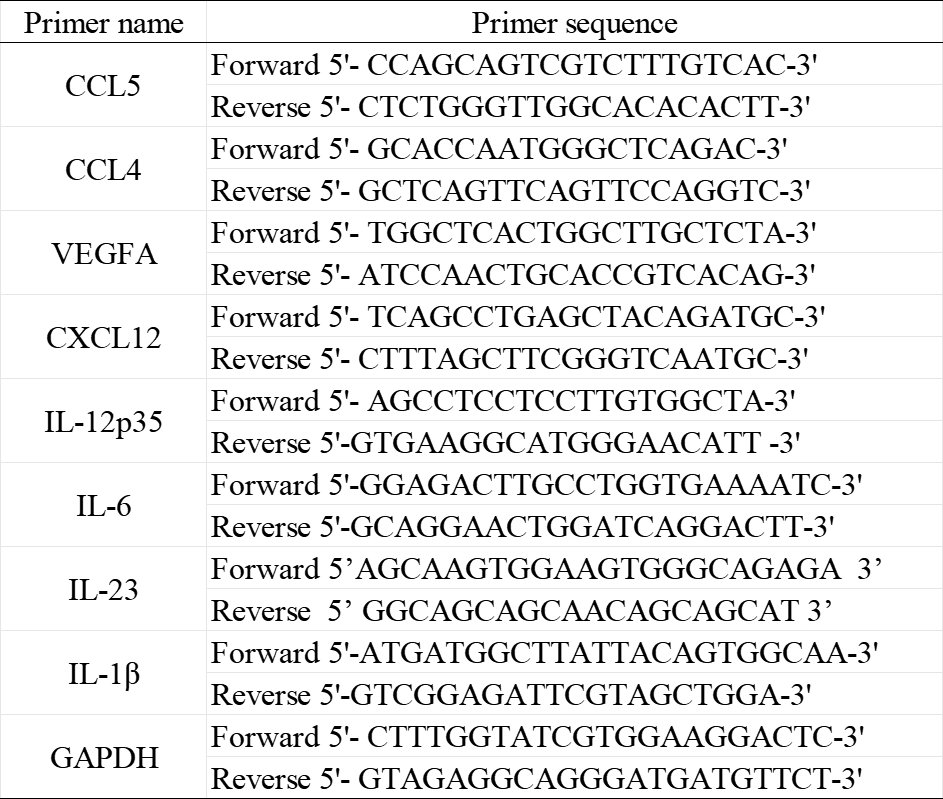


**Table S3. Clinicopathological factors in 35 patients with oral lichen planus**


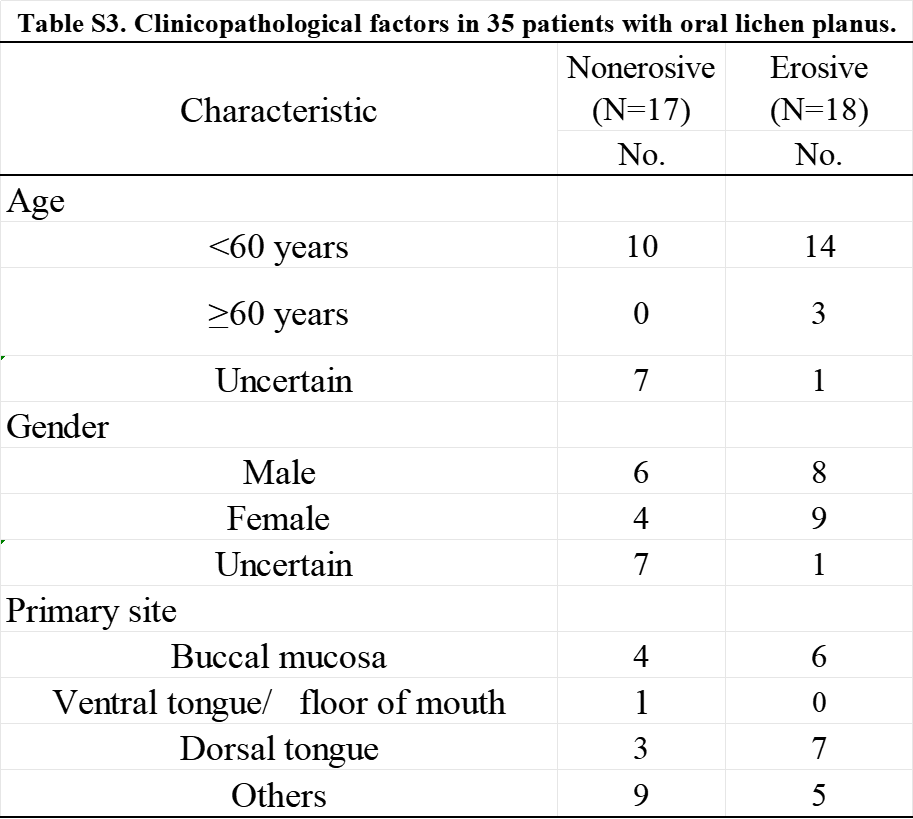

Supplement: Supplementary file 1 — Supporting Information [file MCO2-5-e561-s002.docx]
